# Neural Correlates of Associative Memory in the Elderly: A Resting-State Functional MRI Study

**DOI:** 10.1155/2015/129180

**Published:** 2015-06-09

**Authors:** Weicong Ren, Rui Li, Zhiwei Zheng, Juan Li

**Affiliations:** ^1^Center on Aging Psychology, Key Laboratory of Mental Health, Institute of Psychology, Chinese Academy of Sciences, Beijing 100101, China; ^2^University of Chinese Academy of Sciences, Beijing 100101, China; ^3^Magnetic Resonance Imaging Research Center, Institute of Psychology, Chinese Academy of Sciences, Beijing 100101, China; ^4^State Key Laboratory of Brain and Cognitive Science, Institute of Biophysics, Chinese Academy of Sciences, Beijing 100101, China

## Abstract

The neural correlates of associative memory in healthy older adults were investigated by examining the correlation of associative memory performance with spontaneous brain oscillations. Eighty healthy older adults underwent a resting-state functional MRI and took a paired-associative learning test (PALT). Correlations between the amplitude of low-frequency fluctuations (ALFF) as well as fractional ALFF (fALFF) in the whole brain and PALT scores were calculated. We found that spontaneous activity as indexed by both ALFF and fALFF in the parahippocampal gyrus (PHG) was significantly positively correlated with associative memory performance, suggesting that the PHG plays a critical role in associative memory in older people.

## 1. Introduction

Associative memory demonstrates a greater decline in older adults compared with item memory [1, 2]. It has been well evidenced that healthy aging is associated with cognitive decline in various domains, especially in episodic memory. A salient feature of age-related differences in episodic memory is the difficulty in creating and retrieving associations between single units of information (associative memory), while memory for individual items is less affected [3]. In other words, associative memory declines earlier than item memory as one gets older. It is important to figure out the neural correlates of associative memory in the older adults for comprehensively understanding the mechanism of cognitive decline with aging. Associative memory is typically assessed using paired-associative paradigms [4].

Many neuroimaging and lesion studies have explored the neuroanatomical underpinnings of associative memory and have suggested that the medial temporal lobe (MTL), lateral parietal cortex, and prefrontal cortex (PFC) play important roles in remembering associations. These regions are involved in binding and retrieving item-item or item-context associations or controlling and monitoring these processes [5–8].

Accumulating evidence suggests that, in older adults, several brain regions, particularly the MTL and PFC, show structural volumetric decreases and functional activation changes [9–11]. It has been demonstrated that aging differentially affects the brain substrates of memory, with the PFC being the most vulnerable, the hippocampus being moderately vulnerable, and the entorhinal cortex being relatively spared [12–14]. White matter connections of the frontal and temporal cortex as well as the frontal-subcortical white matter tracts also have been found to play critical roles in age-related differences in associative memory performance [15]. The structural morphology and functional changes in these regions may be the underlying reasons for the age-related decline in associative memory.

Resting-state functional magnetic resonance imaging (fMRI) is useful for exploring the spontaneous functional architecture of the brain [16–18]. In addition, it provides a straightforward comparison of brain activity across different cognitive ability groups. Low-frequency fluctuations observed on resting-state fMRI are physiologically meaningful [16], which are thought to reflect spontaneous regional neuronal activity [19] and different physiological states of the brain [20]. The resting-state amplitude of low-frequency fluctuations (ALFF) can predict task-evoked brain activation [21, 22], as well as correlate with behavioral performance [23–25]. Therefore, the ALFF has been used to examine synchrony in healthy adults [26, 27] and disease-related changes in brain activity [28]. To improve the sensitivity and specificity in detecting spontaneous brain activities, fractional ALFF (fALFF) that is calculated as the ratio of power spectrum of low-frequency to that of the entire frequency range has also been introduced in resting-state fMRI studies [29].

In the present study, we focused on the relationship between the resting-state ALFF and associative memory in healthy older adults. In addition, fALFF was also employed to comprehensively investigate the relationship between oscillations and associative memory. The verbal paired-associative learning test (PALT) was used to assess associative memory [30]. To be specific, the objective of this study was to identify the neural correlates of associative memory in healthy older adults by examining correlations between the resting-state ALFF/fALFF and performance on PALT. Since the MTL and PFC play important roles in associative memory processes and are vulnerable to aging, we postulated that the oscillations in these two regions might be significantly correlated with PALT performance.

## 2. Materials and Methods

### 2.1. Participants

The participants were recruited via advertisements posted at communities near the Institute of Psychology, Chinese Academy of Sciences in Beijing. After baseline evaluation, we enrolled 80 healthy older volunteers who met the following criteria: (1) age ⩾ 60 years; (2) education ⩾ 6 years; (3) a score of ⩾22 on the Beijing Version of the Montreal Cognitive Assessment (MoCA) [31]; and (4) no neurological and psychiatric disorders and traumatic brain injury. No participant was excluded and finally data from 80 participants (43 female, mean age = 70.29 years, range 60–80 years; mean years of education = 14.40 years, range 6–20 years) were analyzed.

This study was approved by the Institutional Review Board of the Institute of Psychology, Chinese Academy of Science. Written informed consent was supplied to all participants and they were paid for their participation. The study was registered in the Chinese Clinical Trial Registry (ChiCTR) (http://www.chictr.org/): ChiCTR-PNRC-13003813.

### 2.2. Memory Task

Associative memory performance was examined using the PALT. For this test, the participants first studied 12 word pairs of nouns, half of which consisted of six semantically related word pairs (e.g., sun-moon), and this was considered as easy condition. The other word pairs included six semantically unrelated word pairs (e.g., teacher-railway), which was considered as difficult condition. After the study session, participants were asked to complete a cued recall task in which the first word of the pair was provided and they had to recall the other paired word. A correctly recalled word was scored 0.5 in the easy condition and 1 in the difficult condition, with the total score for the test equal to 9.

### 2.3. Data Acquisition

A 3.0-Tesla Siemens Trio scanner (Erlangen, Germany) was used for image acquisition at Beijing MRI Center for Brain Research. During the scan, the participants were placed in a supine position with their heads held snugly by a belt and foam pads. They were required to keep their eyes closed, relax, and keep their heads still but not to fall asleep during the scan. For each participant, functional images were collected using an echo-planar imaging sequence. The imaging parameters were repetition time (TR) = 2000 ms; echo time (TE) = 30 ms; flip angle = 90°; field of view (FOV) = 200 mm × 200 mm; slice thickness = 3.0 mm; gap = 0.6 mm; acquisition matrix = 64 × 64; in-plane resolution = 3.125 × 3.125; 33 axial slices; and 200 volumes.

Additionally, a high-resolution structural T1-weighted magnetization-prepared rapid gradient-echo image was also collected for each subject with the parameters as follows: 176 slices; acquisition matrix = 256 × 256; voxel size = 1 mm × 1 mm × 1 mm; TR = 1900 ms; TE = 2.2 ms; and flip angle = 9°.

### 2.4. Resting-State fMRI Data Processing and Statistics

Resting-state fMRI data analyses were performed using the Data Processing Assistant for Resting-State fMRI (DPARSF V2.2) Basic Edition, Statistical Parametric Mapping program (SPM8), and the Resting-State fMRI Data Analysis Toolkit (REST V1.8).

*Preprocessing.* The first ten volumes were discarded for signal equilibrium and participant's adaptation to scanning noise. The rest of the volumes were corrected for intravolume acquisition time delay between slices and intervolume geometrical displacement due to head movement. All functional data were normalized to the Montreal Neurological Institute (MNI) space with 3 × 3 × 3 mm^3^ resampling. Spatial smoothing with a 4-mm Gaussian kernel and linear detrending were finally performed. No participant included in this study exhibited head motion of more than 2.0 mm in any direction or 2.0° rotation throughout the resting-state scans.

*ALFF/fALFF Calculation.* The ALFF value of each voxel was measured as the sum of amplitudes within the low-frequency range [32]. For each voxel, the time series was first converted to the frequency domain using a Fast Fourier Transform (FFT) analysis to obtain the power spectrum. The power spectrum was obtained by square-rooted FFT and averaged across 0.01–0.08 Hz at each voxel. The averaged square root was taken as the ALFF. To reduce global effects of variability across the participants, the ALFF value of each voxel was divided by the global mean ALFF value. For fALFF, the measure was calculated as the ratio of power of low-frequency fluctuations (0.01–0.08 Hz) to that of all available frequencies.

*ALFF/fALFF-PALT Correlation.* To find the neural correlates of associative memory indexed by resting-state ALFF/fALFF, we performed correlation analyses between PALT scores and ALFF/fALFF in the whole brain. At each voxel, the Pearson correlation coefficient between ALFF/fALFF and PALT scores across participants was calculated, with age, gender, and years of education as covariates. Clusters were considered as significant at the combined voxel extent threshold of uncorrected *P* < 0.01 and cluster extent > 486 mm^3^, as determined based on AlphaSim correction by Monte Carlo simulation to *P* < 0.05 (single voxel *P* < 0.01, and spatial smoothness = 4 mm).

## 3. Results

### 3.1. Demographic and Neuropsychological Results

Demographic information and neuropsychological scores are shown in Table 1. MoCA scores ranged from 22 to 30, with an average of 26.76 ± 2.34. PALT scores ranged from 0 to 7 with an average of 2.94 ± 1.47.

### 3.2. Correlations between PALT Scores and the ALFF/fALFF in the Whole Brain

**Figure 1:**
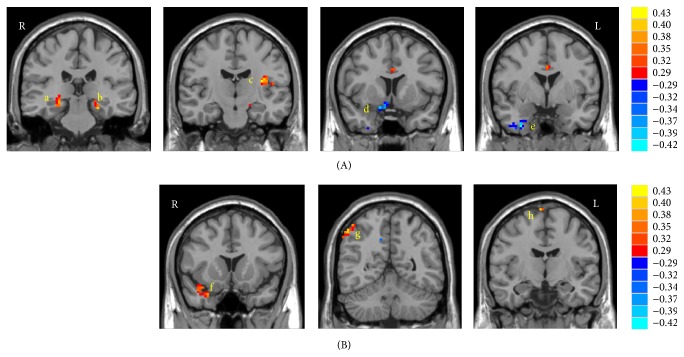
Regional oscillations and associative memory correlation analyses. (A) Statistical map for the correlations between performance on the PALT and ALFF in the bilateral PHG (a, b), left insula (c), right IFG (d), and right ITG (e). (B) Statistical map for the correlations between performance on the PALT and fractional ALFF in the right PHG/STG (f), right IPL (g), and right SMA/SFG (h). The correlation values are indicated using the color scales on the right.

Voxelwise correlation analyses showed that resting-state ALFF values correlated with PALT scores in a number of brain regions (Figure 1(A), Table 2). Specifically, the ALFF values positively correlated with PALT scores in the bilateral parahippocampal gyri (PHG) and left insula, while negative correlations were found between them in the right inferior temporal gyrus (ITG) and right inferior frontal gyrus (IFG).

In the correlation analyses between fALFF values and PALT scores, fALFF in three regions showed significant positive correlation with PALT performance including the right PHG/superior temporal gyrus (STG), right inferior parietal lobule (IPL), and right supplementary motor area (SMA)/superior frontal gyrus (SFG) (Figure 1(B), Table 2).

**Figure 2:**
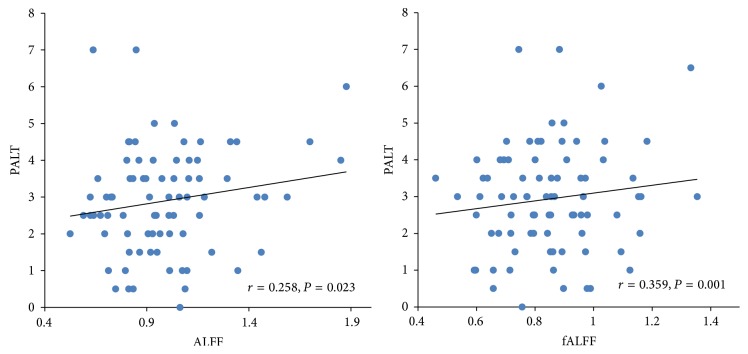
Scatter plots of the significant relationship between PALT scores and ALFF as well as fALFF values in the right PHG with age, gender, and education as covariates. Each dot represents data from one participant.

As the results of the correlation analyses, spontaneous activity in the right PHG was demonstrated to be robustly correlated with associative memory performance assessed by PALT. Scatter plots of PALT scores versus ALFF/fALFF values were displayed to illustrate the relationship between spontaneous oscillations in the right PHG and PALT scores with age, education, and gender taken as covariates (Figure 2). ALFF/fALFF values under/over 3 standard deviations away from the mean value of the sample were identified as outliers and removed from the data set. Thus the ALFF value from one participant was removed during analyses. The results demonstrated that PALT scores were positively correlated with ALFF (*r* = 0.258, *P* = 0.023) and fALFF (*r* = 0.359, *P* = 0.001) in the right PHG.

## 4. Discussion

The objective of this study was to investigate the neural correlates of associative memory in healthy older adults using resting-state fMRI. Results showed that spontaneous activity indexed by both ALFF and fALFF in the right PHG was significantly positively correlated with associative memory performance, suggesting a critical role of the right PHG in associative memory in older adults.

There is compelling evidence that the MTL, consisting of the hippocampal region and the adjacent perirhinal, entorhinal, and parahippocampal cortices, plays an important role in associative memory [33]. It has been shown that age-related shrinkage occurs in the MTL of healthy adults [13]. As the principal neocortical input pathway to the hippocampal region [34], the PHG is thought to function in memory formation [35]. Düzel et al. reported that the PHG was involved in the visual associative recognition memory for spatial and nonspatial stimulus configurations [36]. Recently, Bar and colleagues also found that the PHG was involved in contextual associations processing [37]. They found that PHG responds more strongly to the rich associations condition compared with the less associations condition. The present findings of significant correlations between ALFF/fALFF in the PHG and associative performance further provided the evidence that the PHG plays an important role in the memory of associations. It suggested that participants with higher regional spontaneous activity in the right PHG performed better in associative memory test, when age, years of education, and gender were controlled. Previous studies have found that both item-spatial context associations and item-nonspatial context associations activated the parahippocampal cortex more than noncontextual items, which indicated that the PHG functions importantly in processing of contextual associations [38]. The present finding further suggested that the PHG may be a pivotal region underlying association memory process.

The two measurements of ALFF and fALFF are highly related but not entirely the same [39]. In the correlation analyses between ALFF and PALT scores, positive correlations were found in the bilateral PHG and left insula, and negative correlations were found in the right ITG and right IFG. Using fALFF as a measurement, only significant positive correlations were found. In addition to a significant correlation between performance on PALT and fALFF in the right PHG, the significant correlations also appeared in the right IPL and right SMA/SFG, suggesting that these regions may also be involved in the network underlying memory process.

Insula was demonstrated to be involved in processing general cognition by the meta-analysis of neuroimaging literatures [40]. Activation in the insula has been shown to be associated with contextual binding of semantic relations and successful encoding for relation load [41, 42]. The parietal cortex also contributed importantly to episodic memory retrieval [43]. Wanger et al. showed that multiple parietal regions were activated during episodic retrieval, including regions within the intraparietal sulcus extending laterally to the IPL. It is also interesting that ALFF in two regions located in ITG and IFC negatively correlated with individual PALT performance. Increased activity in the lateral temporal and superior frontal regions has been previously found in AD and MCI patients. Besides, AD patients also showed increased activity in the ITG as compared with MCI patients [28, 44]. Here the negative correlation between PALT scores and the ALFF in the right ITG and right IFG indicated that participants with lower performance in associative memory test may have higher ALFF value in these regions, similar to the results in the disease studies. Thus the result suggested that, in addition to PHG playing a crucial role, the insula and some cortical regions especially the frontal and parietal regions may also be involved in associative memory in the elderly. It would be interesting in future studies to investigate how the PHG cooperates with other regions in the cortex to function in associative memory in older adults.

Finally it is methodologically important to note that as the ALFF is more prone to noise from physiological sources than fALFF, we could not rule out the potential effect of physiological noise on the ALFF results [32]. The ALFF-PALT results need to be examined in independent samples. In addition, the individual-level standardization in the ALFF/fALFF analysis used in the present study might lose the individual differences in the group analysis, and a group-level standardized procedure is required to validate these results in future studies [45].

## 5. Conclusion

In the present study, we explored the neural correlates of associative memory by resting-state ALFF as well as fALFF. Our results showed that the PHG may be critically involved in associative memory in older adults, and participants with higher regional spontaneous activity in the right PHG performed better in associative memory test.

## Figures and Tables

**Table 1:** Demographic characteristics and neuropsychological results of participants.

	Participants (*N* = 80)
Age	70.29 ± 5.64
Education (y)	14.40 ± 3.11
Female/male	43/37
MoCA	26.76 ± 2.34
PALT	2.94 ± 1.47

Note: MoCA: Montreal Cognitive Assessment; PALT: paired-associative learning test.

**Table 2:** Peaks of regions showing significant ALFF/fALFF-PALT correlations.

Measurement	Regions	BA	Number of voxels	Peak MNI coordinates		
				*x*	*y*	*z*
ALFF	PHG (L)	35/36	24	−24	−27	−18
	PHG (R)	35/36	27	24	−27	−9
	Insula (L)	13	28	−33	−15	15
	ITG (R)	20	40	33	0	−42
	IFG (R)	47	35	15	9	−18

fALFF	PHG/STG (R)	38	33	30	12	−30
	IPL (R)	40	25	54	−54	48
	SFG/SMA (R)	6	21	6	−9	78

Note: MNI coordinates of the center of gravity of each cluster. PHG: parahippocampal gyrus; ITG: inferior temporal gyrus; IFG: inferior frontal gyrus; STG: superior temporal gyrus; IPL: inferior parietal lobule; SFG: superior frontal gyrus; SMA: supplementary motor area; L: left hemisphere; R: right hemisphere.
